# Self DNA from Lymphocytes That Have Undergone Activation-Induced Cell Death Enhances Murine B Cell Proliferation and Antibody Production

**DOI:** 10.1371/journal.pone.0109095

**Published:** 2014-10-08

**Authors:** Qing Lu, Ji-Yang Wang, Luman Wang, Xuechao Jiang, Yiwei Chu

**Affiliations:** 1 Key Laboratory of Medical Molecular Virology of MOE/MOH, Department of Immunology, School of Basic Medical Sciences, Fudan University, Shanghai, China; 2 Biotherapy Research Center of Fudan University, Shanghai, China; INSERM-Université Paris-Sud, France

## Abstract

Systemic lupus erythematosus (SLE) is characterized by prominent autoinflammatory tissue damage associated with impaired removal of dying cells and DNA. Self DNA-containing immune complexes are able to activate both innate and adaptive immune responses and play an important role in the maintenance and exacerbation of autoimmunity in SLE. In this study, we used DNA from lymphocytes that have undergone activation-induced cell death (ALD-DNA) and analyzed its role on the activation and differentiation of B cells from normal BALB/c mice as well as lupus-prone MRL^+/+^ and MRL/lpr mice. We found that ALD-DNA directly increased the expression of costimulatory molecules and the survival of naïve B cells *in vitro*. Although ALD-DNA alone had little effect on the proliferation of naïve B cells, it enhanced LPS-activated B cell proliferation *in vitro* and *in vivo*. In addition, ALD-DNA increased plasma cell numbers and IgG production in LPS-stimulated cultures of naïve B cells, in part via enhancing IL-6 production. Importantly, B cells from lupus mice were hyperresponsive to ALD-DNA and/or LPS relative to normal control B cells in terminal plasma cell differentiation, as evidenced by increases in CD138^+^ cell numbers, IgM production, and mRNA levels of B lymphocyte-induced maturation protein-1 (Blimp-1) and the X-box binding protein 1 (XBP1). Furthermore, ALD-DNA enhanced CD40-activated naïve B cell proliferation. Collectively, these data indicate that self DNA can serve as a DAMP (damage-associated molecular pattern) that cooperates with signals from both innate and adaptive immunity to promote polyclonal B cell activation, a common characteristic of autoimmune diseases.

## Introduction

Systemic lupus Erythematosus (SLE) is a chronic-relapsing autoimmune disease of incompletely understood etiology and is characterized by polyclonal B cell activation and autoantibodies to components of the cell nucleus, such as DNA and the nucleosome, a complex composed of 180 bp of DNA and five histone molecules. These autoantibodies may induce inflammation and tissue damage, most prominently glomerulonephritis through immune complex deposition.

Autoimmunity results from the failure of tolerance to autoantigens. Although the origin of anti-dsDNA antibodies is at present controversial, exposure of the immune system to endogenous DNA can lead to the development of autoimmunity, as demonstrated by the finding that insufficient digestion of extracellular DNA in DNase I deficient mice causes SLE-like disease [1] and that mutations of *DNASE I* have been identified in humans with SLE [2]. In SLE patients and murine lupus, excessive apoptosis with a defect in clearance of apoptotic cells is implicated as one source of extracellular DNA [3]–[6]. Furthermore, DNA-containing immune complexes (ICs) in serum of SLE patients were shown to activate plasmacytoid dendritic cells to overproduce type I IFN and the serum type I IFN levels correlated with both SLE disease activity and severity [7], [8].

A direct correlation was established between endogenous DNA and autoantibody production in studies with transgenic AM14 B cells specific for autologous IgG2a (rheumatoid factor, RF). ICs containing IgG2a mAbs specific for DNA or chromatins can directly activate autoreactive AM14 RF^+^ B cells to proliferate in a T-cell independent (TI) manner by dual engagement of the B cell receptor (BCR) and intracellular Toll-like receptor (TLR) 9. DNA component in antigen is a critical factor for these immunostimulatory ICs to activate autoreactive AM14 RF^+^ B cells [9]. TLR9 was first shown to uniquely recognize unmethylated CpG motif rich in microbial DNA and transmit mitogenic signals to B cells, although it was subsequently shown that TLR9 might also mediate mammalian DNA recognition. It has been proposed that the endosomal localization of nucleic acid-sensing TLRs may be an evolutionary strategy to protect them from access to self nucleic acids [10], [11]. Thus, DNA containing ICs are actively involved in anti-nucleic acid and RF autoantibody production, and in the maintenance and exacerbation of autoimmunity [12].

B cells play an important role in protective immunity by producing large amounts of antibodies against invading pathogens. B cells are also responsible for the development and pathogenesis of both systemic and organ-specific autoimmune diseases, as highlighted by the clinical efficacy of B-cell depletion therapies [13], [14]. B cells sense antigens through antigen-specific BCRs and innate pattern recognition receptors (PRRs) such as TLRs. In general, the antibody response against thymus dependent protein antigens (TD-Ags) requires the antigen-specific CD4^+^ T helper cells, which provide help for antigen specific B-cell activation via CD40-CD40L interactions and by cytokines in the germinal centers (GCs). Here, activated B cells proliferate and undergo class switch recombination (CSR), affinity maturation, and differentiate into memory B cells or high affinity antibody-secreting plasma cells. The TI antibody response can be elicited by microbial products in the absence of helper T cells. Both LPS (TLR4 ligand) and unmethylated CpG DNA (TLR9 ligand) can trigger polyclonal activation of naïve mouse B cells and induce proliferation and differentiation into short-lived plasma cells [15]. However, human naïve B cells express low to undetectable levels of TLRs, and therefore require prior stimulation via BCR to respond to TLR ligands (microbial products) irrespective of the nature of T helper cells present [16]. In contrast to naïve B cells, human memory B cells have higher constitutively expressed TLRs and can respond directly to TLR stimulation to induce B cell proliferation and differentiation into plasma cells [16], [17]. Requirement of cognate T cell help is usually a constraint for autoreactive B cell activation. The finding that microbial products such as LPS or CpG DNA could circumvent this control may explain at least in part the well-known association between infections and the flare or onset of autoantibody-mediated diseases [18].

A series of studies have revealed that injecting SLE-non-susceptible BALB/c mice with DNA from lymphocytes that have undergone activation-induced cell death (ALD-DNA) mixed with bacterial adjuvants could evoke SLE-like autoimmune diseases [19], [20]. Indeed, DNA trapped in the plasma ICs from SLE patients showed size laddering and low molecular weight that were characteristic of the apoptotic process [21], [22]. To better understand the immune properties of self DNA and its role in initiation and progression of B-cell autoimmunity, we used DNA from lymphocytes that had undergone activation-induced cell death (ALD-DNA) to directly stimulate B cells from normal BALB/c and two murine lupus-like models, MRL^+/+^ and MRL/lpr. MRL^+/+^ mice uniformly acquire lupus like disease, but disease is relatively mild and progression is slow. The genes predisposing to autoimmunity have not yet been clearly identified. The *lpr* mutation of *Fas* on the MRL genetic background accelerates and exacerbates disease in MRL/lpr mice [23]. Our results indicated that ALD-DNA cooperated with LPS in promoting B cell activation under normal and autoimmune conditions. Importantly, B cells from lupus mice showed heightened responsiveness to ALD-DNA and/or LPS in the terminal plasma cell differentiation and antibody production. Furthermore, ALD-DNA enhanced the proliferation of anti-CD40-activated naïve B cells. Taken together, our results indicate that self DNA can serve as a DAMP (damage-associated molecular pattern) that cooperates with signals from both innate and adaptive immunity to promote polyclonal B cell activation, a common characteristic of autoimmune diseases.

## Materials and Methods

### Ethics Statement

This work was conducted according to the guidelines for care of animals used for scientific purposes (Ministry of Health, P.R. China, 1998) and with the ethical approval of the Shanghai Medical Laboratory Animal Care and Use Committee (Permit number: 2009–0082) as well as the Ethical Committee of Fudan University (Permit number: 20120002). All experiments carried out in this study were strictly performed in a manner to minimize suffering of laboratory mice.

### Mice

Female BALB/c, lupus-prone MRL/lpr, and nude mice were purchased from the Shanghai laboratory Animal Center (SLAC), Chinese Academy of Science (Shanghai, P. R. China). Female lupus-prone MRL^+/+^ mice were kindly provided by Dr. Nan Shen, Joint Molecular Rheumatology Laboratory, Shanghai Ren Ji Hospital and Institute of Health Sciences, Shanghai JiaoTong University, Shanghai, China. All mice were housed in the animal facility of Fudan University under specific pathogen free conditions. BALB/c and nude mice used in this study were aged between 6 weeks to 8 weeks, whereas MRL^+/+^ and MRL/lpr mice were sacrificed at 12-week-old and 16- to 20-week-old, respectively.

### Lymphocyte purification and culture conditions

Single-cell suspensions were prepared by crushing spleens between frosted glass slides. Splenic resting B cells from BALB/c and MRL^+/+^ mice were isolated by negative selection using anti-CD43-coated magnetic beads (Miltenyi Biotech, Bergisch Gladbach, Germany). The cell isolation was performed according to the manufacturer's protocol. Splenic B cells from MRL/lpr mice were obtained by positive selection using anti-CD19 MicroBeads (Miltenyi Biotech) according to the manufacturer's protocol. High purity of cells(≥98%) was confirmed by performing flow cytometric analysis. Purified B cells were cultured in RPMI 1640 medium supplemented with 10% FCS, 50 µM 2-mercaptoethanol, and penicillin/streptomycin (100 U/ml). Unless indicated otherwise, the concentrations of LPS (Sigma-Aldrich, St. Louis, MO), ALD-DNA, and anti-CD40 antibody (eBioscience, San Diego, CA) used for stimulation were 100 ng/ml, 50 µg/ml, and 1 µg/ml, respectively.

### ALD-DNA preparation

Splenocytes from female BALB/c mice were cultured for 6 days in the presence of 5 µg/ml concanavalin A (Con A) (Sigma-Aldrich) to trigger activation-induced cell death (AICD). DNA was purified from Con A-activated splenocytes by extraction with phenol-chloroform-isoamyl alcohol (25/24/1) and ethanol precipitation followed by repeated extraction with Triton X-114 to remove endotoxin. The level of endotoxin was monitored using the Limulus amebocyte lysate assay (detection limit 0.1 EU/ml, BioWhittaker, Walkersville, MD, USA). The endotoxin levels before and after Triton X-114 extraction were 10.4 EU/mg DNA and <1 EU/mg DNA, respectively. The DNA concentration was determined by measuring absorbance at 260 nm. The A260/A280 ratio for all the DNA preparations was ≥1.9. The apoptotic DNA laddering was confirmed by performing agarose gel electrophoresis (Figure S1A). Endotoxin-free *Escherichia coli* genomic DNA was purchased from InvivoGen (San Diego, CA). Single-stranded (ss) DNA was prepared by boiling dsDNA for 10 min followed by immediate cooling on ice for 5 min.

### B cell proliferation assay

Cell proliferation was analyzed by CFSE dilution assay or BrdU incorporation. For CFSE dilution assay, B cells were incubated with 1 µM CFSE (eBioscience) for 10 min at room temperature, and then washed with medium containing 10% FCS. CFSE-labeled B cells were cultured with ALD-DNA, LPS, *E. coli* DNA, ALD-DNA plus LPS, or *E. coli* DNA plus LPS for 3 days. The cells were analyzed using a CyAn ADP analyzer (Beckman-Coulter) and the Modfit software (Verity Software House, Topsham, ME).

For BrdU incorporation, ALD-DNA, anti-CD40 antibody, LPS, or a combination of these stimuli were added to the cultures for 3 days. In some cases, cells were pretreated with 20 µg/ml polymyxin B (PMB, Sigma-Aldrich) for 20 min at 37°C. Cells were harvested after 24-hour incubation with 10 µM BrdU (Roche Diagnostics, Mannheim, Germany). BrdU incorporation was analyzed using Cell proliferation ELISA Kit (Roche Diagnostics) according to the manufacturer's instruction.

### *In vivo* assay of B cell proliferation

Nude mice were administered an intravenous injection containing either 50 µg ALD-DNA, or 1 µg LPS, or a combination of both dissolved in 200 µl PBS on day 1. An additional intraperitoneal injection of BrdU (2 mg; BD Biosciences, San Diego, CA) was administered 24 h before sacrificing the mice. Mice that were intravenously injected with sterile PBS were used to determine background values. After 3 days, spleens were collected and BrdU incorporation assay was performed using a FITC BrdU Flow kit (BD Pharmingen, San Diego, CA) according to the manufacturer's protocol.

### Antibodies and flow cytometric analysis

Cells stimulated with ALD-DNA, LPS, or a combination of these stimuli for 3 days were stained with B220-APC (RA3-6B2, eBioscience), CD86-PE (GL1, eBioscience), MHC class II-FITC (2G9, BD Biosciences), or CD138-PE (281-2, BD Biosciences). CFSE-labeled B cells stimulated with ALD-DNA, *E. coli* DNA, LPS, anti-CD40, or different combinations of these stimuli for 3 days were stained with B220-APC (RA3-6B2, eBioscience) or CD138-APC (281-2, BD Biosciences). Appropriate isotype-matched antibodies were included as controls. Data were acquired on a CyAn ADP Analyzer and analyzed using Summit 5.2 software (both from Beckman-coulter). Dead cells and debris were excluded by forward scatter and side scatter (FSC/SSC) gating and propidium iodide staining (Sigma-Aldrich). For BrdU incorporation assay using a FITC BrdU Flow kit, cells were fixed, permeabilized, and treated with DNase prior to BrdU staining using a FITC-coupled anti-BrdU monoclonal antibody, as recommended by the manufacturer. The incorporated BrdU was measured in conjunction with DNA content by 7-AAD staining.

### Detection of IL-6, IL-10, and antibodies by ELISA

To analyze antibody production, purified splenic B cells were cultured in ALD-DNA, LPS, or ALD-DNA plus LPS. The culture supernatant was collected from day 6 cultures, and the antibody levels were measured using Mouse IgM ELISA Ready-SET-Go (detection range, 0.39–25.00 ng/ml) for IgM and Mouse IgG ELISA Ready-SET-Go (detection range, 1.56–100.00 ng/ml) for IgG. For cytokine production, purified splenic B cells were cultured with ALD-DNA, LPS, *E. coli* DNA, a combination of ALD-DNA and LPS, or a combination of *E. coli* DNA and LPS for 72 h. Then supernatant was assayed using Mouse IL-6 ELISA Ready-SET-Go (detection range, 4–500 pg/ml) for IL-6 and Mouse IL-10 ELISA Ready-SET-Go (detection range, 32–4000 pg/ml) for IL-10, respectively. All kits were purchased from eBioscience and experimental procedures were performed following the manufacturer's instructions.

### Analysis of Blimp-1, XBP1 and IL-6 mRNA expression by real-time PCR

Purified splenic B cells were cultured in the presence of LPS, ALD-DNA, or a combination of both for 6 h to induce IL-6, and 4 d to induce Blimp-1 and XBP1 expression. Total RNA was extracted using RNeasy Mini kit (Qiagen, Valencia, CA) and cDNA was generated using PrimeScript RT reagent Kit (TaKaRa, Dalian, China) as per manufacturer's instructions. Gene expression was analyzed by real-time PCR using the SYBR Premix Ex Taq (TaKaRa), according to manufacturer's instructions. The primer pairs used for real-time PCR were as follows: 5′-TGC TTA AAA CTC CAT GAC CTC-3′ and 5′-GCT ACA CAC CCT CAC CTC TG-3′ for Blimp-1; 5′-CAG GTG GAC ATG GGA TTC-3′ and 5′-TGC ACA TAA GGG AAA ACA AG-3′ for XBP1; 5′-TCC AAT GCT CTC CTA ACA GA-3′ and 5′-ACT AGG TTT GCC GAG TAG ATC-3′ for IL-6; 5′-ATG CTC CCC GGG CTG TAT-3′ and 5′-CAT AGG AGT CCT TCT GAC CCA TTC-3′for β-actin. All reactions were performed in triplicate in an ABI PRISM 7700 system and purity of the amplified PCR products was determined by a heat-dissociation protocol to enable detection of non-specific amplification. Target gene expression in each sample was calculated by the comparative Ct method using β-actin for normalization, and expressed relative to the untreated sample.

### Statistical analysis

*P* values were determined by using the paired t test for *in vitro* experiments and a Mann-Whitney U-test (2-tailed Student's t-test using non parametric criteria for independent samples) for *in vivo* experiments. All analyses were performed using GraphPad Prism software. A value of *P*<0.05 was considered indicative of a significant difference.

## Results

### ALD-DNA enhances LPS induced survival and proliferation of naïve B cells

**Figure 1:**
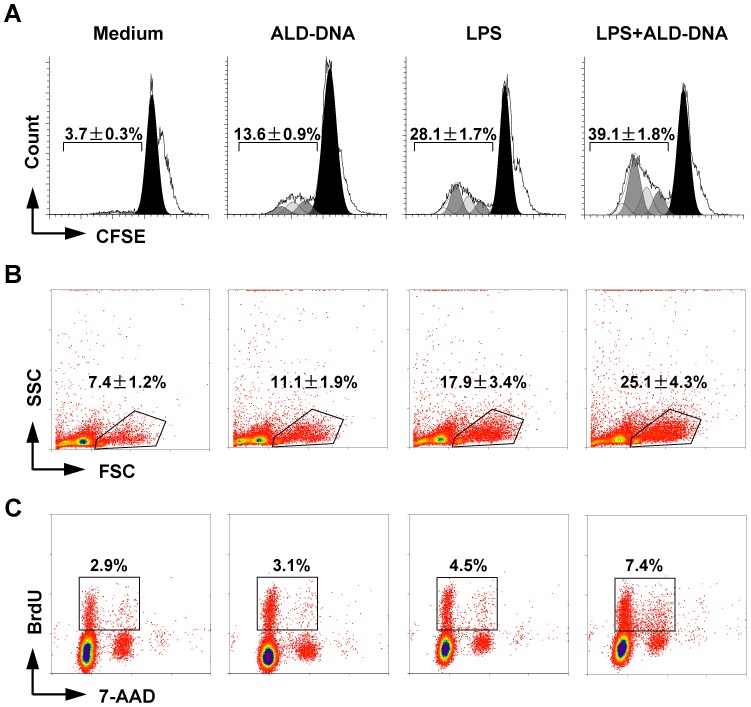
Enhancement of LPS-stimulated B cell survival and division by ALD-DNA. (**A**) CFSE-labeled B cells were stimulated with ALD-DNA (50 µg/ml) in the presence or absence of LPS (100 ng/ml) for 72 h. Division progression of B cells was determined by dilution of CFSE. Representative histogram plots show the percentage of proliferating (CFSE-low) B cells after each treatment (mean values ±SEM, n = 6). Shaded areas show the cell division peaks predicted by the ModFit software. (**B**) Flow cytometry dot plots showing the gate used to select living cells and eliminate cell debris, and showing the viability of B cells (mean values ±SEM, n = 11) under different culture conditions as described above for 72 h. (**C**) Nude mice were administered one intravenous injection of indicated stimuli at day 1 and an additional intraperitoneal injection of BrdU 24 h before sacrifice. After 72 h, spleens were collected, and B220^+^ B cells with incorporated BrdU combined with total DNA content (with 7-AAD) were analyzed by FACS. The region gates applied to the 7-AAD versus BrdU dot plot indicate the cell subsets that were resided in the S phase of the cell cycle. Results represent 6 mice/experimental group from two separate experiments.

Initial experiments in which splenic resting B cells were stimulated with a range of ALD-DNA concentrations (25–100 µg/ml) revealed a weak but dose-dependent proliferative effect of ALD-DNA. We then examined whether ALD-DNA was able to collaborate with TLR4-derived signals and enhance LPS-induced B cell proliferation. The CFSE assay data indicated that ALD-DNA (25 µg/ml–100 µg/ml) cooperated with low doses of LPS to induce B cell proliferation (Figure S1B). The observed effects were additive or approximately additive and became less evident as the doses of LPS were increased, suggesting that the ALD-DNA-mediated enhancement was prominent on suboptimally activated B cells (Figure S1C). We further confirmed the cooperation of ALD-DNA and LPS for B cell survival and proliferation by performing additional *in vitro* and *in vivo* experiments. The flow cytometry analyses indicated that ALD-DNA alone enhanced the cell division (Figure 1A, *P* = 0.0002) and survival (Figure 1B, *P* = 0.01) of naïve B cells. In addition, ALD-DNA further enhanced LPS-induced proliferation (Figure 1A, *P* = 0.0003) and survival (Figure 1B, *P* = 0.0002). Analysis of the CFSE histograms revealed that the percentage of B cells that underwent four rounds of division was considerably higher in the presence of both ALD-DNA and LPS (39.1%) than in the presence of ALD-DNA (13.6%) or LPS (28.1%) alone (Figure 1A). The percentage of gated live cells in cell culture samples stimulated by both ALD-DNA and LPS (25.1%) was considerably higher than those for cells stimulated with ALD-DNA (11.1%) or LPS (17.9%), or for control cells (7.4%) (Figure 1B). To investigate whether ALD-DNA was able to enhance LPS induced B cell proliferation in a TI-manner *in vivo*, we injected ALD-DNA, LPS or both into nude mice and analyzed cell division by BrdU incorporation. Consistent with the results of *in vitro* experiments, we found a significant increase in the BrdU^+^ proliferating B cells after injecting both ALD-DNA and LPS (7.4%) as compared with ALD-DNA (3.1%) or LPS (4.5%) alone (Figure 1C, n = 6, *P*<0.05). These data indicate that ALD-DNA and LPS are able to promote B cell proliferation both under *in vitro* and *in vivo* conditions.

### ALD-DNA directly induces CD86 and MHC class II expression on B cells

**Figure 2:**
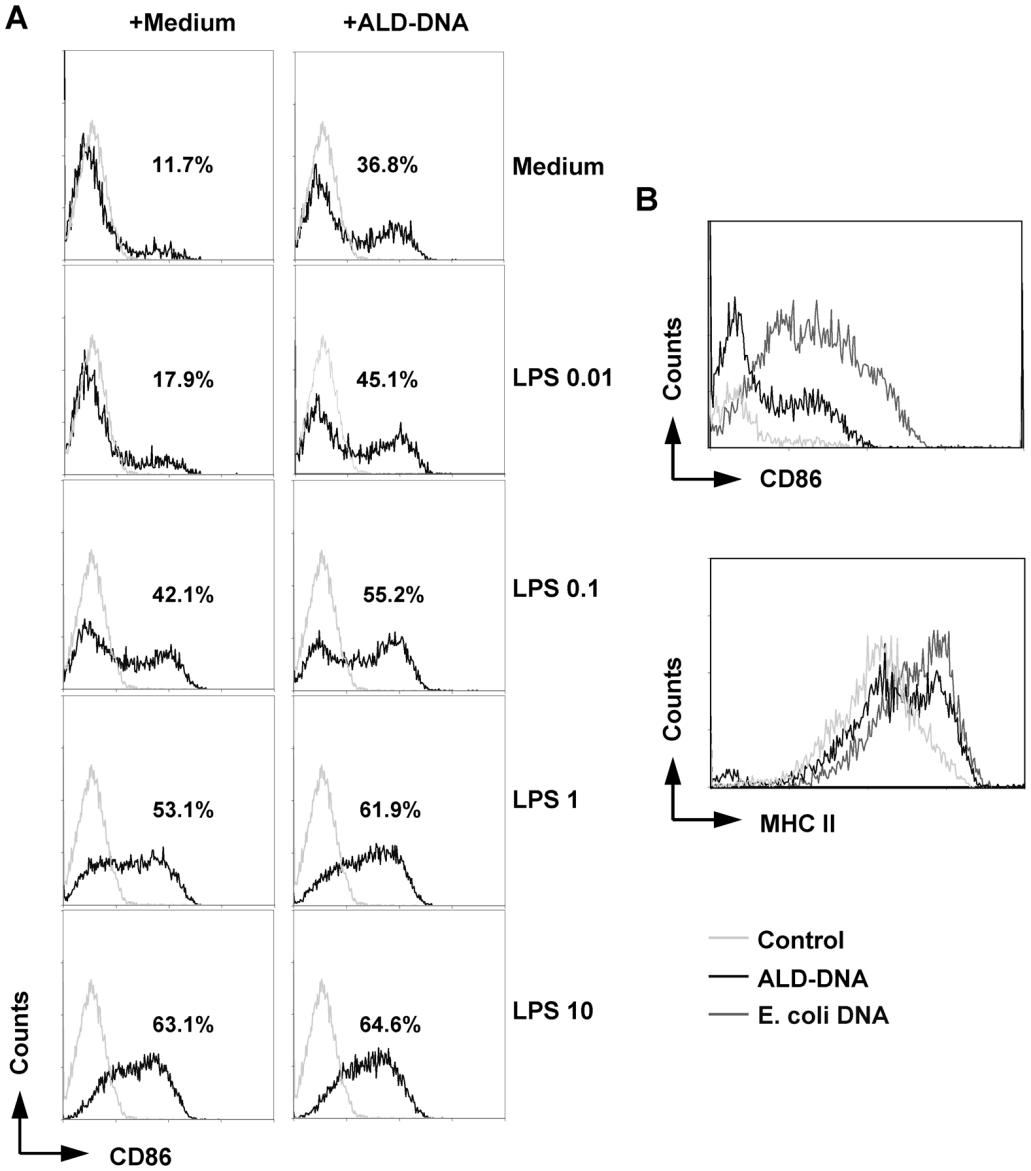
Up-regulation of CD86 and MHC class II expression in naïve B cells by ALD-DNA stimulation. (**A**) Naïve B cells were incubated with ALD-DNA (50 µg/ml) in the presence or absence of LPS ranging from 0.01 µg/mL to 10 µg/ml for 72 h. The numbers of CD86-expressing B cells after each treatment were determined by flow cytometry. (**B**) Naive B cells were stimulated with ALD-DNA or *E. coli* single-stranded (ss) DNA (both at 50 µg/ml) for 72 h. CD86 and MHC class II expression was determined by performing flow cytometry. Histogram plots show the mean fluorescence intensity (MFI) of these surface molecules. The data are representative of three independent experiments.

B cell activation is often accompanied by up-regulation of MHC class II and CD86 expression, which facilitate antigen presentation to and activation of helper T cells. We therefore analyzed the effect of ALD-DNA on the expression of these molecules. As shown in the left panels of Figure 2A, LPS up-regulated CD86 expression in a dose-dependent manner. ALD-DNA stimulation increased the percentage of CD86^+^ B cells to 36.8% as compared with the control cells that were stimulated with medium alone (11.7%; top two panels of Figure 2A). Furthermore, addition of ALD-DNA resulted in an increase in CD86 expression induced by LPS at suboptimal concentrations (Figure 2A, right panels). ALD-DNA also up-regulated MHC class II expression, although the effect was not as strong as that induced by bacterial DNA (Figure 2B). These results indicate that ALD-DNA can directly stimulate B cells to up-regulate CD86 and MHC class II expression, however, the immunostimulatory effect is less potent than that induced by bacterial DNA.

### ALD-DNA and LPS synergize for inducing IgG production in naive B cells

**Figure 3:**
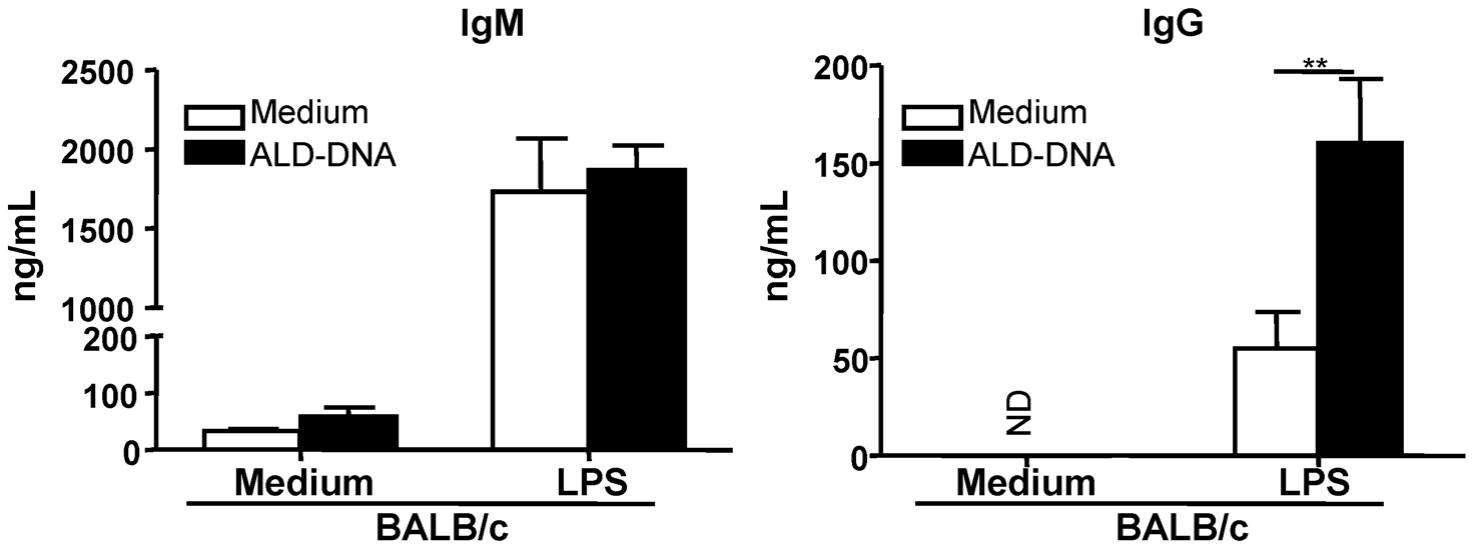
Synergistic enhancement of LPS-driven IgG production in naive B cells by ALD-DNA stimulation. Naive B cells were cultured for 6 days in media containing ALD-DNA (50 µg/ml) with or without LPS (100 ng/ml). Cell culture supernatants were collected, and the levels of IgM and IgG were measured by ELISA. Bars represent the means ±SEM of three independent experiments (n = 5). ND (not detected). **P<0.01.

After demonstrating that ALD-DNA enhanced LPS-induced survival, proliferation and the up-regulation of CD86 expression, we next analyzed the effect of ALD-DNA on the antibody production by LPS-activated B cells. LPS stimulation preferentially increased the production of IgM (Figure 3, lift panel). Addition of ALD-DNA did not further increase LPS-induced IgM production. However, LPS-induced IgG production was significantly higher (Figure 3, right panel, *P* = 0.007) with ALD-DNA stimulation (IgG yield: 160.5±32.6 ng/ml; n = 5) as compared with that of cell cultures stimulated with LPS alone (IgG yield: 55.4±18.6 ng/ml; n = 5).

### Effect of ALD-DNA on LPS-driven plasma cell differentiation in naïve B cells

**Figure 4:**
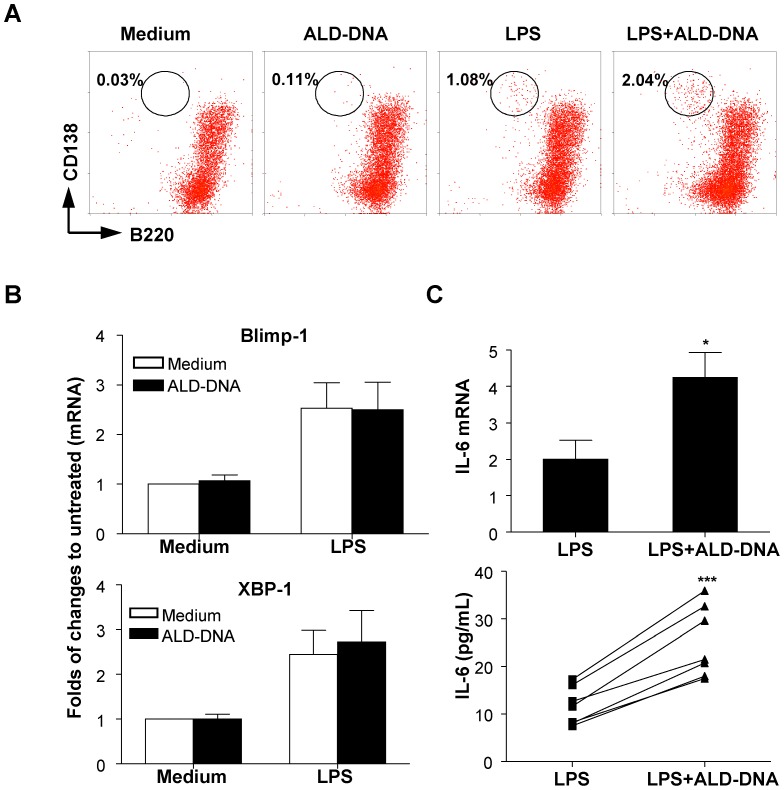
Effect of ALD-DNA on CD138, Blimp-1, XBP1, and IL-6 expression in naïve B cells. Naïve B cells were cultured in media containing ALD-DNA (50 µg/ml), LPS (100 ng/ml), or both for different periods as described below. (**A**) After 72 h stimulation, Cells were analyzed for B220 and CD138 surface expression by flow cytometry. The number indicates the percentage of B220^−^CD138^+^ cells in the gate. Similar results were obtained in three independent experiments. (**B**) Expression of Blimp-1 and XBP1 mRNA from B cells cultured for 96 hours was examined by real-time PCR. Fold induction relative to their untreated controls is shown (means ±SEM, n = 5). (**C**) Expression of IL-6 mRNA in B cells after 6 h stimulation was examined by real-time PCR. Fold induction relative to their untreated controls is shown (means ±SEM, n = 5); The amounts of IL-6 protein in the culture supernatants of B cells after 72 h stimulation were measured by ELISA (n = 6, a line linked two dots represents a pair observation). Up-regulation of IL-6 protein was undetectable in all samples after ALD-DNA treatment. *P<0.05 as compared to the LPS, ***P<0.001 as compared to the LPS.

We next analyzed the effect of ALD-DNA on the generation of CD138^+^ plasma cells. Stimulation with a suboptimal dose of LPS resulted in the induction of a small proportion of B220^−^CD138^+^ cells (1.08%; Figure 4A). Addition of ALD-DNA to LPS-stimulated culture led to about 1.9-fold increase in this population (2.04%; Figure 4A). Molecular events that control plasma cell differentiation include expression of the transcription factors Blimp-1 and XBP1 [24]. ALD-DNA did not up-regulate Blimp-1 and XBP1 mRNA expression in naïve B cells (Figure 4B). In addition, ALD-DNA did not enhance LPS-induced Blimp-1 and XBP1 mRNA expression (Figure 4B). Therefore, although ALD-DNA increased the proportion of LPS-induced plasma cells, it did not seem to affect the expression of transcription factors that played essential roles in plasma cell differentiation.

We assessed whether ALD-DNA and LPS together enhance the survival of plasma cells. IL-6 has been shown to be a potent *in vitro* and *in vivo* growth factor for murine plasmacytosis [25], [26]. In addition, IL-6 as a nonswitching factor enhanced IgG production in IgG-committed B cells of a particular subclass [27]. We therefore measured IL-6 mRNA and protein expression after 6 h and 3 days of cultures, respectively. ALD-DNA enhanced the LPS-induced IL-6 mRNA transcription (Figure 4C, upper panel, *P* = 0.02) and protein secretion (Figure 4C, lower panel, *P* = 0.0001) by naïve B cells. These data indicate that ALD-DNA is able to increase the proportion of plasma cells and IgG production in LPS stimulated cultures in part by enhancing the secretion of IL-6.

### ALD-DNA and LPS cooperate to stimulate B cells from lupus mice

**Figure 5:**
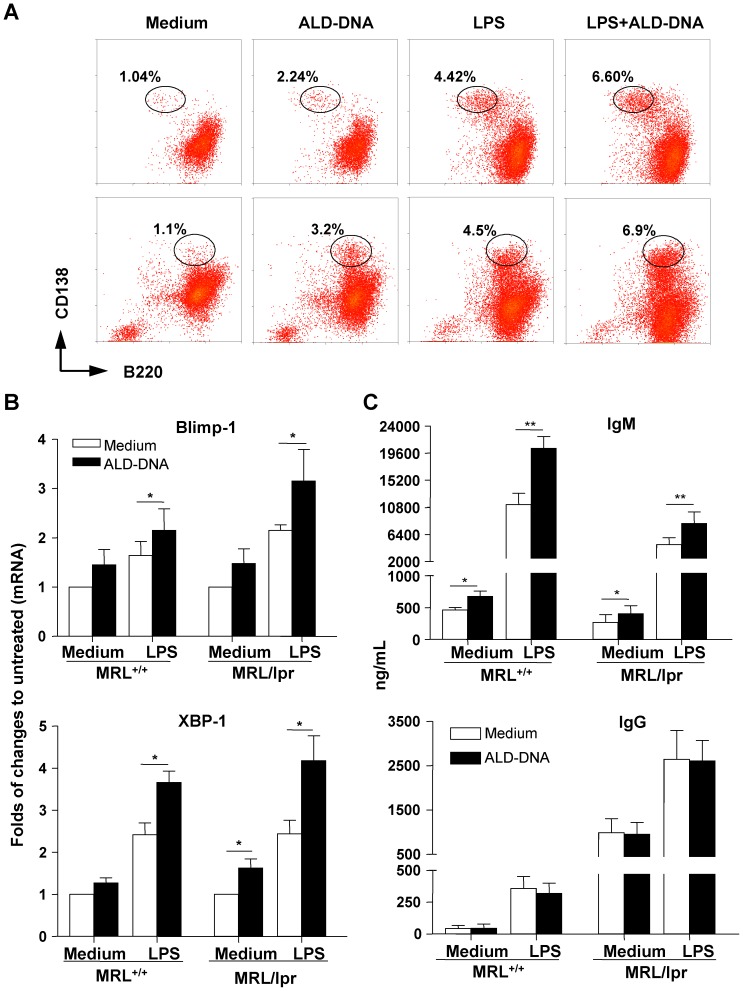
Promotion of plasma cell differentiation and Ig production in lupus B cells by ALD-DNA. Splenic B cells from 16- to 20-week-old MRL/lpr mice and 12-week-old MRL^+/+^ mice were cultured in media alone, or in the presence of ALD-DNA (50 µg/ml), LPS (100 ng/ml), or both for different periods as indicated below. (**A**) After 72 h stimulation, viable cells were analyzed for B220 and CD138 surface expression by flow cytometry. Representative dot plots showed the frequencies of plasmablasts (B220^+^CD138^hi^, lower panels, MRL/lpr, n = 6) or plasma cells (B220^−^CD138^+^, upper panels, MRL^+/+^, n = 6) generated after each treatment. (**B**) Expression of Blimp-1 and XBP1 mRNA from lupus B cells cultured for 96 h was examined by real-time PCR. Results are expressed as the fold induction of gene transcription relative to their untreated controls (means ±SEM, n = 6). (**C**) Ig production in cell culture supernatants harvested on day 6 was determined by ELISA. Bars represent the means ±SEM of three independent experiments (n = 6). *P<0.05, **P<0.01.

To assess the cooperative effect of LPS and ALD-DNA under autoimmune conditions, splenic B cells isolated from lupus-prone MRL^+/+^ and MRL/lpr mice that had developed a lupus-like disease were stimulated with ALD-DNA and/or LPS *in vitro*. As shown in Figure 5A (lower panels, MRL/lpr),ALD-DNA alone induced a 2.9-fold increase in the number of B220^+^CD138^+^ plasmablasts over those enumerated in the control. LPS induced a higher number of B220^+^CD138^+^ plasmablasts (4.5%), which were further enhanced by the addition of ALD-DNA (6.9%). LPS also induced viable cell numbers (36.1%) that were at least twice as high as those induced by ALD-DNA stimulation (16.2%), as determined by flow cytometry gated on FSC/SSC plots (data not shown). B cells from MRL^+/+^ mice responded to ALD-DNA and/or LPS stimulation by generation of the B220^−^CD138^+^ plasma cells (Figure 5A, upper panels), in contrast to the generation of the B220^+^CD138^+^ plasmablasts by MRL/lpr B cells (Figure 5A, lower panels). Therefore, B cells from MRL/lpr and MRL^+/+^ mice both responded to ALD-DNA and LPS to undergo B cell terminal differentiation although these cells were driven to different stages of plasma cell generation.

The differentiation of activated B cells into plasma cells is regulated by transcriptional cell programs. Consistent with the findings of increased percentages of CD138^+^ cells after ALD stimulation, the real-time PCR analysis revealed that ALD-DNA stimulation significantly increased XBP1 and to a lesser extent, Blimp-1 mRNA expression in LPS stimulated lupus B cells (Figure 5B, *P*<0.05%) but not normal BALB/c B cells (Figure 4B). ALD-DNA alone also caused slight increases in XBP1 and Blimp-1 mRNA expression in lupus B cells.

The effect of ALD-DNA and/or LPS on antibody production by lupus B cells was also investigated (Figure 5C). ALD-DNA induced a similar 1.5-fold increase in IgM production in both MRL^+/+^ and MRL/lpr B cells as compared to their unstimulated controls. B cells from two lupus mice showed robust response to LPS stimulation and produced high amount of IgM as compared to normal BALB/c mice (Figure 5C and Figure 3). The presence of ALD-DNA markedly enhanced LPS-induced IgM production by lupus B cells (Figure 5C, upper panel) but not normal BALB/c B cells (Figure 3, lift panel). It is intriguing to note that MRL/lpr B cells produced lower amount of IgM than did MRL^+/+^ B cells in response to ALD-DNA and/or LPS (Figure 5C, upper panel). In contrast, MRL/lpr B cells produced greater amount of IgG, even in the absence of stimulation, than did MRL^+/+^ B cells. ALD-DNA did not further enhance LPS-induced IgG production by either MRL^+/+^ or MRL/lpr B cells (Figure 5C, lower panel). Collectively, ALD-DNA and LPS together promoted the differentiation of plasma cells and the production of IgM but not IgG by lupus B cells.

### Cooperation between ALD-DNA and CD40 signal

**Figure 6:**
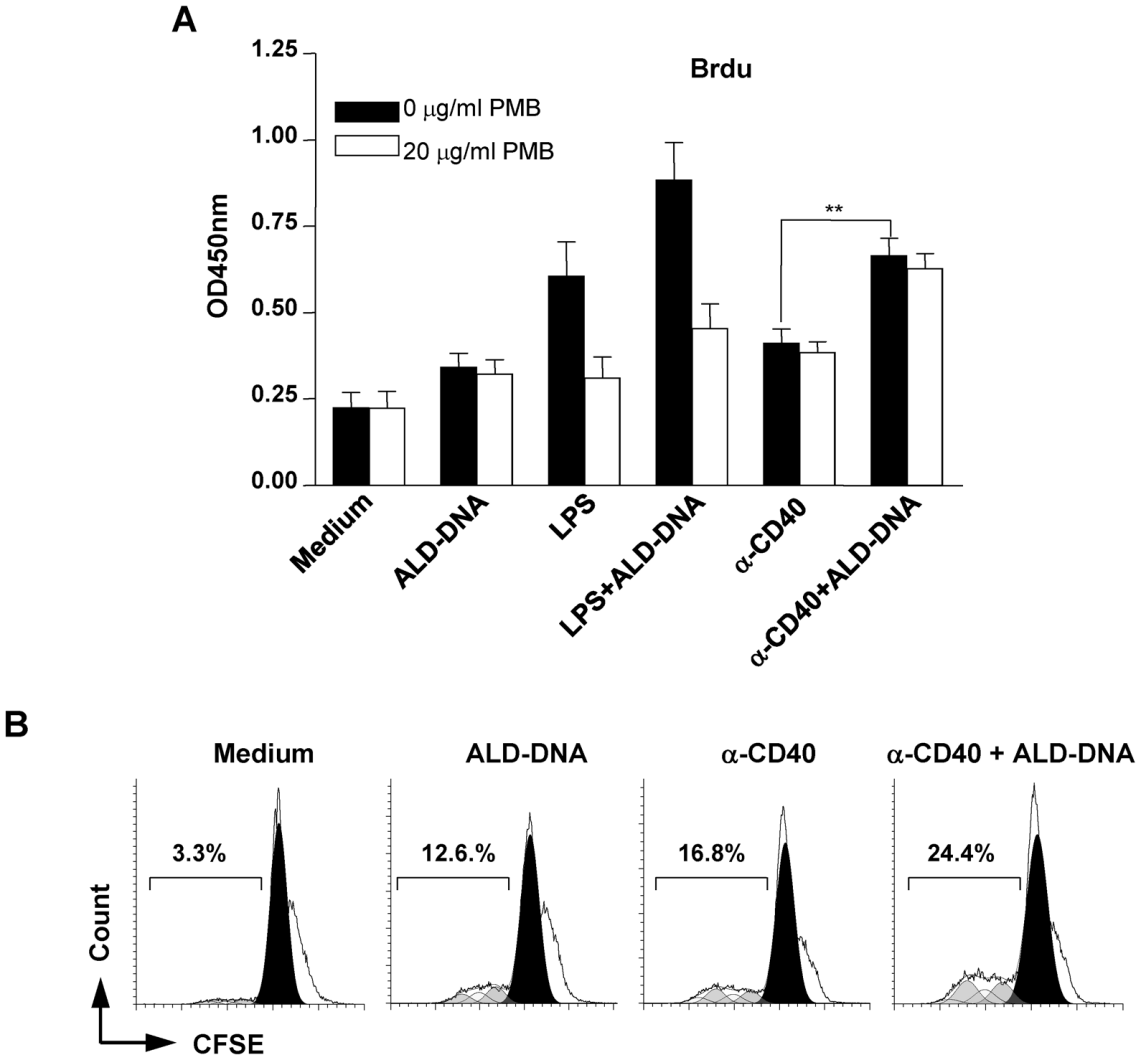
Enhancement of CD40-stimulated naïve B cell proliferation by ALD-DNA. (**A**) Naïve B cells were pre-incubated for 20 min with polymyxin B (PMB; 20 µg/ml) before the addition of ALD-DNA (50 µg/ml), LPS (100 ng/ml), anti-CD40 (1 µg/ml), LPS (100 ng/ml) plus ALD-DNA (50 µg/ml), or anti-CD40 (1 µg/ml) plus ALD-DNA (50 µg/ml). After 72 h stimulation, cell proliferation was detected by performing BrdU incorporation assay using ELISA. Data are presented as the means ±SEM of three independent experiments. (**B**) CFSE-labeled naïve B cells were cultured in media containing ALD-DNA, anti-CD40, or both (at the concentrations as described above) for 72 h. Cell division was monitored by measuring the dilution of CFSE. Shaded areas show the cell division peaks predicted by the ModFit software. Results from one representative experiment of three are shown. **P<0.01.

We have thus far provided evidence that ALD-DNA can cooperate with LPS to activate B cells obtained from normal and autoimmune mice. To investigate whether ALD-DNA can also cooperate with signals from helper T cells, we analyzed the effect of ALD-DNA on B cell proliferation induced by CD40 ligation. As shown in Figure 6A, ALD-DNA caused a significant increase in BrdU incorporation by naive B cells stimulated with an anti-CD40 antibody. The increased BrdU incorporation might result from accelerated cell cycle progression, elevated cell survival, or both. To distinguish these possibilities, we monitored cell division by performing CFSE dilution assay. About 1.45-fold more cells underwent four rounds of cell divisions in the presence of anti-CD40 antibody plus ALD-DNA as compared with cells stimulated with anti-CD40 antibody alone (Figure 6B). The enhancement of ALD-DNA on CD40-stimulated naïve B cell proliferation was not blocked by LPS inhibitor PMB (Figure 6A), indicating that the observed effect of ALD-DNA was not mediated by endotoxin contamination. Taken together, these results show that self DNA may serve as an endogenous adjuvant in CD40-mediated B cell response.

## Discussion

Excessive apoptosis with deficiency in clearance of apoptotic cells was related to the extracellular source of DNA in patients with SLE as well as murine lupus. Additionally, primary lymphocyte necrosis occurred in patients with SLE [28], [29], and SLE is characterized by prominent inflammatory tissue damage. DAMPs including genomic and mitochondrial DNA, RNA, histones, and high mobility group box 1 (HMGB1), all which are described as non-infectious endogenous TLR ligands, may be released into circulation or within tissues causing and accelerating inflammation [30]–[34]. Ishii et al. reported that genomic DNA released from dying cells stimulated DC maturation and boosted both antigen specific antibody and CD8^+^ T-cell immune response [35]. However, while bacterial genomic DNA triggered direct B-cell activation, vertebrate genomic DNA did not, and the difference in CpG status was considered to be the cause of the mitogenicity of bacterial DNA [36].

In the current study, we found that ALD-DNA alone or in the presence of suboptimal doses of LPS enhanced the survival and proliferation of B cells and increased expression of MHC class II and CD86 molecules in naïve B cells. Moreover, ALD-DNA increased a number of plasma cells and IgG production in LPS stimulated cultures in part by enhancing the secretion of IL-6. To our knowledge, this is the first study to demonstrate that mammalian DNA from apoptotic cells can directly stimulate normal naïve B cells and modulate their function.

While we found that ALD-DNA promoted LPS-triggered proliferation of naïve B cells, Dil and Marshall reported that CpG ODN and LPS did not synergistically induce naïve B cell proliferation, but significantly stimulated IL-10 production [37]. Furthermore, microbial product-induced IL-10 production appears to mitigate exaggerated B cell responses to pathogenic microbes by potently inhibiting CpG- or LPS-induced B cell proliferation [16], [38]. Therefore, CpG ODN and LPS are not synergy in B cell proliferation due to the synergistic induction of large amounts of IL-10 [37]. Consistent with these findings, we observed that bacterial genomic DNA also greatly enhanced LPS-triggered IL-10 production (Figure S2C), but to a lesser extent than CpG ODN [37]. Bacterial DNA and LPS had neither additive nor synergetic effect on naïve B cell proliferation, although both stimulants were potent mitogens for B cells (Figure S2A). In addition, the immunosuppressive effect of IL-10 on CpG ODN-stimulated Ig-secreting cell generation was also previously reported [16]. Similarly, we found that stimulation with bacterial DNA along with LPS did not enhance differentiation into plasma cells (Figure S2B). In contrast, stimulation with ALD-DNA alone had no effect on IL-10 expression and slightly increased LPS-triggered IL-10 production (Figure S3). Taken together, the data indicate that ALD-DNA is functionally distinct from CpG ODN and bacterial genomic DNA, and is able to enhance LPS-mediated proliferation and plasma cell generation in part due to its weak ability to enhance LPS-triggered IL-10 production.

Remarkably, we found that ALD-DNA treated lupus B cells were able to induce surface CD138 and secrete IgM (Figure 5). ALD-DNA slightly increased XBP1 and Blimp-1 mRNA expression. These data suggested that ALD-DNA at least partially activated plasma cell differentiation and promoted a subset of lupus B cells to become IgM-secreting plasmablasts/plasma cells. In contrast, ALD-DNA alone had little effect on the increases in CD138^+^ cell numbers, antibody production and mRNA levels of XBP1 and Blimp-1 in normal naïve B cells (Figure 3 and 4). Therefore, lupus B cells appear to be more sensitive to ALD-DNA stimulation than normal B cells. Furthermore, ALD-DNA enhanced LPS-triggered plasmablast/plasma cell differentiation program of lupus B cells, as evidenced by increases in CD138^+^ cell numbers, XBP1 and Blimp-1 mRNA expression as well as IgM production (Figure 5).

The ELISA data indicated that ALD-DNA enhanced LPS-induced IgG, but not IgM, production in normal naïve B cells (Figure 3). In striking contrast, ALD-DNA promoted LPS-induced IgM, but not IgG, production in lupus B cells (Figure 5). Therefore, ALD-DNA had distinct effect on antibody production in normal and lupus B cells, at least under these *in vitro* culture conditions. IL-6 is a survival factor for plasma cells [25], [26] and acts as a nonswitching factor to enhance IgG production by committed B cells [27]. ALD-DNA promoted LPS-induced IL-6 expression in normal naïve B cells and this finding may in part account for the increased IgG production. Conversely, ALD-DNA did not enhance LPS-induced IL-6 expression (ALD-DNA plus LPS and LPS alone induced IL-6 at concentrations of 59 pg/ml and 63.2 pg/ml, respectively; data not shown) in lupus B cells.

The mechanisms that enable ALD-DNA to selectively stimulate IgM production by lupus B cells, but not normal B cells, remain unclear. Moreover, lupus B cells showed higher responsiveness than normal B cells to ALD-DNA and/or LPS stimulation to undergo terminal differentiation and yielded higher antibody production. These differential effects might be partially attributable to the distinct B cell subset composition. Enlargement of marginal zone (MZ) B cell population was reported in different mouse models of lupus such as MRL^+/+^, MRL/lpr, NZB/W F1, Palmerston North (PN) and BAFF transgenic mice [39]–[41]. Our result also confirmed this abnormality (Figure S4). MZ B cells are programmed for efficient differentiation into mature plasma cells with the ability to secrete massive quantities of IgM in response to TLR agonists such as LPS [42]. In addition, MZ B cells have an enhanced secretory apparatus [43] and lower activation thresholds [44]. These unique capacity might allow MZ B cells to respond to TLR ligands that occur at low concentrations and have low-affinity, including less favorable CpG sequences found in hypomethylated mammalian DNA [40]. Additionally, we used CD19 microbeads to purify splenic B cells from MRL/lpr mice that had actively developed a lupus-like disease, in order to avoid loss of any B cell subpopulations. Thus B cells isolated from 16-week-old MRL/lpr mice might have a cell population shift toward antigen-activated B cells, antibody producing plasmablasts [45], and memory B cells, in contrast to predominantly resting B cells isolated from normal BALB/c and prediseased MRL^+/+^ mice. Therefore the differential responsiveness to ALD-DNA and/or LPS stimulation cannot be simply ascribed to distinct cell activation states *in vivo*.

Normal MZ B-cell repertoires contain a high frequency of autoreactive B cells including anti-DNA [46]. An expansion of the MZ B-cell subset is common in several genetically distinct murine lupus models, implying a role of MZ B cells involved in disease pathogenesis. Furthermore, MRL/lpr mice have a high frequency of apoptotic lymphocytes in secondary lymphoid organs with a macrophage defect in apoptotic cell phagocytosis [4]. Impaired removal of dying cells resulted in deposition and accumulation of apoptotic DNA in the formation of IgM ICs in the splenic marginal zone [5]. It has been noted that DNA released from necrotic/apoptotic cells complexes with HGMB1 can facilitate the entry of nucleic acids into cells to activate endosomal TLR9 [47]. It is possible that apoptotic DNA when bound to natural IgM in the formation of ICs may increase its availability not only to MZ but also follicular (FO) B cells through engagement of FcμR on the surface of B cells [48], and this may occur at the prediseased stage in lupus prone mice.

Thus, cooperation of self DNA and low levels of LPS represent an attractively cooperative model of DAMPs and PAMPs, and may serve two important roles: firstly, to aid quick and efficient clearance of infections before they become pathogenic, and secondly, in some circumstances, initiate polyclonal B cell response often characteristic of autoimmune diseases either at the origin [49] or in an amplification loop [50]–[52].

We have analyzed the cooperation effects of stimulation with ALD-DNA and LPS, the ligand for TLR4. In contrast to murine B cells, human B cells express TLR1/2, TLR7, TLR9 but not TLR4 [16], [53]. However, terminally differentiated plasma cells isolated from human peripheral blood have been shown to express all TLRs including TLR4 with the exception of TLR10, and their antibody production and secretion can be enhanced after engagement of TLRs [53]. Synergistic activation of B cells by different PRRs like TLR9 and TLR2 [54] or TLR9 and NOD-like receptors (NLRs) [55] has been reported. It would be interesting to explore whether ALD-DNA can cooperate with innate PPR ligands (in addition to TLR4 ligand LPS) that modulate B cell activation, and to compare the responsiveness of B cell subsets as well as plasma cells to these stimuli. Such studies will help us understand the potential roles of self DNA in human B-cell autoimmunity development and aid in designing novel therapeutic strategies for SLE.

Notably, ALD-DNA also enhanced CD40 activated naïve B cell proliferation (Figure 6). GCs are candidate locations where B cells may receive signals from CD40 and apoptotic DNA as well as bacterial products. In GCs of some patients with SLE, tingible body macrophages were defective in uptake of apoptotic cells, and apoptotic nuclear debris was attached on the surfaces of follicular dendritic cells [56], [57]. Activated B cells express CD40 ligand CD154 in both patients with SLE and lupus prone mice [58], [59]. Our results implicated that DNA might enhance CD40 signaling in B cells activated by homotypic CD40-CD154 interactions. It is possible that DNA in conjunction with signaling from either CD40 or PRRs may promote positive selection of autoreactive B cells that are generated by random somatic hypermutation of Ig genes during TD GC reaction or TI extrafollicular response [60]–[62].

In conclusion, the current study has demonstrated that self DNA can serve as a DAMP that cooperates with signals from both innate and adaptive immunity to promote polyclonal B cell activation, a common characteristic of autoimmune diseases. Importantly, B cells from lupus mice showed heightened responses to ALD-DNA and/or LPS in the terminal plasma cell differentiation and antibody production, indicating that self DNA could be a contributing factor to hyperactive B-cell compartments in SLE. Further elucidation of the molecular and cellular mechanisms by which ALD-DNA contributes to B cell activation would provide new insights for the development of novel therapeutic strategies for SLE.

## Supporting Information

Figure S1 **Dose-specific effects of ALD-DNA and/or LPS on naïve B cell proliferation.** (**A**) DNA purified from ConA-activated lymphocytes that had undergone AICD (size standards, lane 1; ALD-DNA, lane 2 and 3). (**B**) CFSE-labeled naïve B cells were stimulated with ALD-DNA (0 µg/mL, 25 µg/mL, 50 µg/mL, or 100 µg/mL) in the presence or absence of 100 ng/ml LPS for 72 h. (**C**) CFSE-labeled naïve B cells were stimulated with LPS (0 µg/mL, 0.01 µg/mL, 0.1 µg/mL, or 1 µg/mL) in the presence or absence of 50 µg/ml ALD-DNA for 72 h. The frequency of proliferating (B220^+^ CFSE-low) B cells (**B and C**) was determined by performing flow cytometry analysis. Data, pooled from three independent experiments, are shown as bar graphs (means ±SEM).
(TIF)

Figure S2 **Effects of bacterial DNA on LPS-induced proliferation, plasma cell generation, and IL-10 production.** (**A**) CFSE-labeled naïve B cells were stimulated with *E. coli* ssDNA (0 µg/mL, 10 µg/mL, 50 µg/mL, or 100 µg/ml) in the presence or absence of 100 ng/ml LPS for 72 hours. The frequency of proliferating (B220^+^CFSE-low) B cells was determined by performing flow cytometry analysis. Data, pooled from three independent experiments, are shown as bar graphs (means ±SEM, n = 5). **P<0.01 as compared to LPS or *E. coli* DNA, and ***P<0.001 as compared to LPS or *E. coli* DNA. (**B**) CFSE-labeled naïve B cells were stimulated with *E. coli* ssDNA (10 µg/mL or 50 µg/ml) in the presence or absence of 100 ng/ml LPS for 72 h. Cells were analyzed by flow cytometry for CD138 surface expression. Representative dot plots of three independent experiments show the percentages of CD138^+^ plasma cells generated under different culture conditions. (**C**) Naïve B cells were cultured in media containing *E. coli* ssDNA (50 µg/ml) with or without LPS (100 ng/ml) for 72 h, and cell culture supernatants were collected for analysis of IL-10 by ELISA. Data, pooled from three independent experiments, are shown as bar graphs (mean ±SEM, n = 4).*P<0.05 as compared to LPS.
(TIF)

Figure S3 **Enhancement of LPS-induced IL-10 production in naïve B cells by ALD-DNA.** Naïve B cells were cultured in media containing ALD-DNA (50 µg/ml) in the presence or absence of LPS (100 ng/ml) for 72 h, and cell culture supernatants were collected for analysis of IL-10 by ELISA. Data, pooled from three independent experiments, are shown as bar graphs (mean ±SEM, n = 4). ND (not detected). *P<0.05 as compared to LPS.
(TIF)

Figure S4 **Frequency of MZ B cells in normal and MRL^+/+^ lupus mice.** Splenic B cells were isolated from normal BALB/c and MRL^+/+^ mice by negative selection using anti-CD43-coated magnetic beads. The frequency of MZ B cells (CD21^hi^ CD23^lo^) was determined by flow cytometry.
(TIF)
