# Supplementary figures and images for: Self DNA from Lymphocytes That Have Undergone Activation-Induced Cell Death Enhances Murine B Cell Proliferation and Antibody Production
Source: PLoS One. 2014 Oct 8;9(10):e109095. doi: 10.1371/journal.pone.0109095 (PMC4189923; doi:10.1371/journal.pone.0109095)

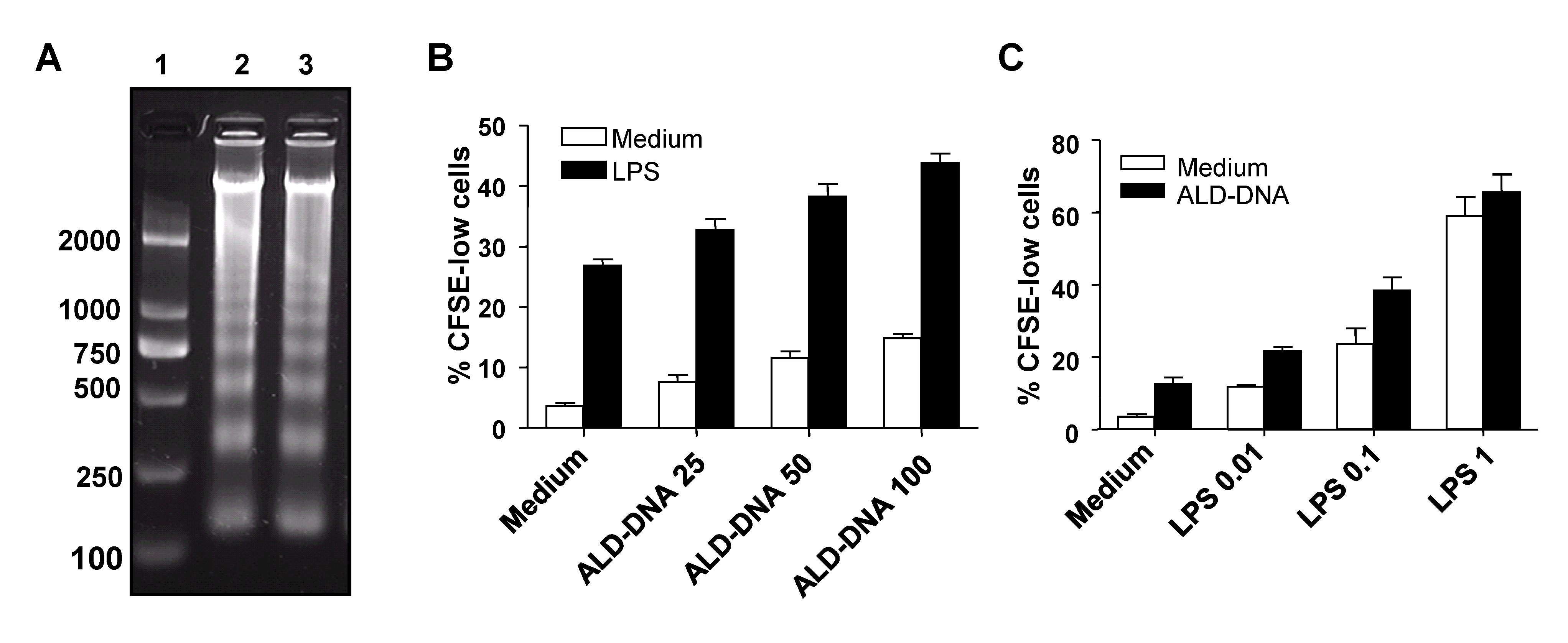

Supplement: Figure S1 — Dose-specific effects of ALD-DNA and/or LPS on naïve B cell proliferation. (A) DNA purified from ConA-activated lymphocytes that had undergone AICD (size standards, lane 1; ALD-DNA, lane 2 and 3). (B) CFSE-labeled naïve B cells were stimulated with ALD-DNA (0 µg/mL, 25 µg/mL, 50 µg/mL, or 100 µg/mL) in the presence or absence of 100 ng/ml LPS for 72 h. (C) CFSE-labeled naïve B cells were stimulated with LPS (0 µg/mL, 0.01 µg/mL, 0.1 µg/mL, or 1 µg/mL) in the presence or absence of 50 µg/ml ALD-DNA for 72 h. The frequency of proliferating (B220+ CFSE-low) B cells (B and C) was determined by performing flow cytometry analysis. Data, pooled from three independent experiments, are shown as bar graphs (means ±SEM). (TIF) [file pone.0109095.s001.tif]

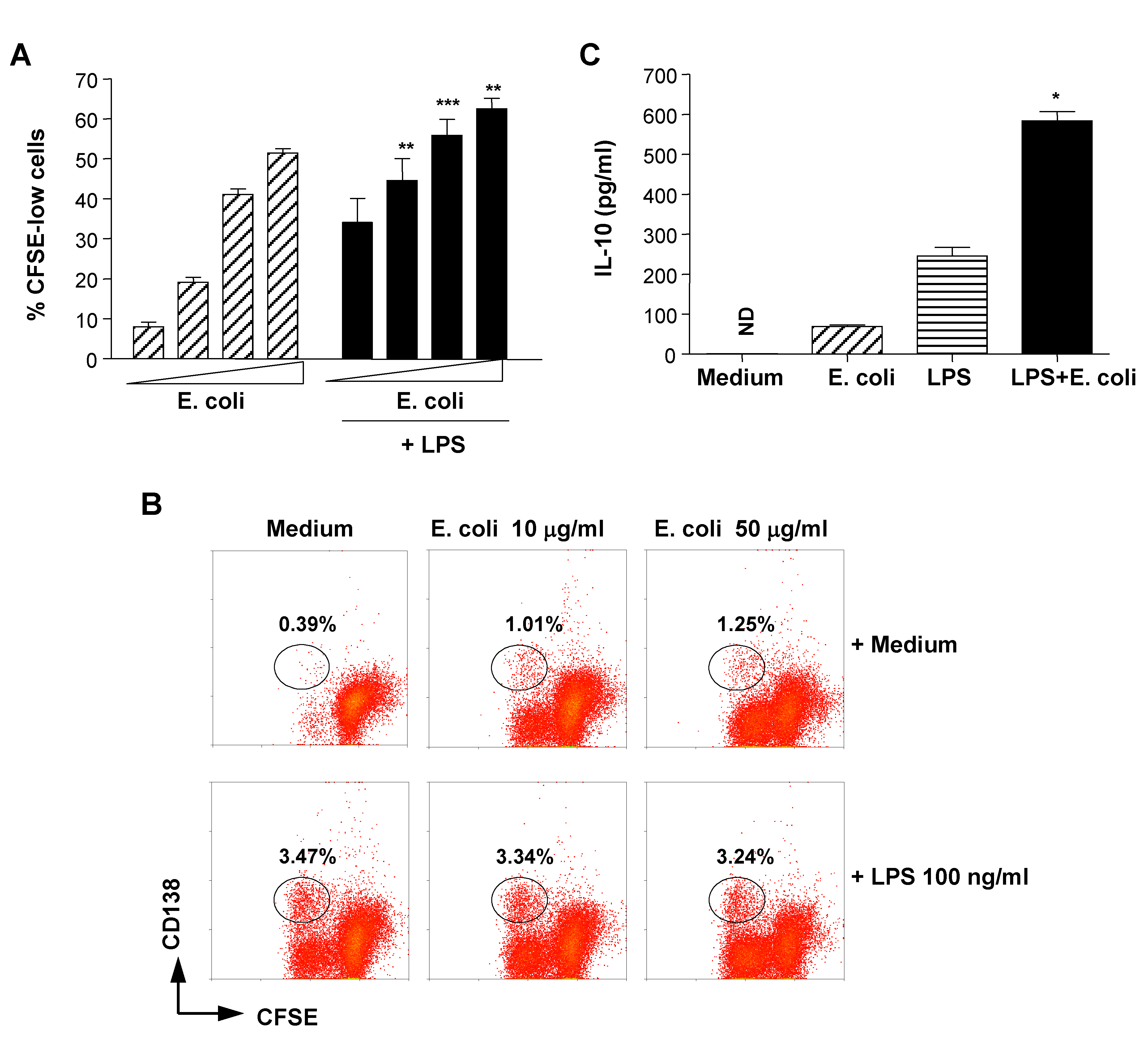

Supplement: Figure S2 — Effects of bacterial DNA on LPS-induced proliferation, plasma cell generation, and IL-10 production. (A) CFSE-labeled naïve B cells were stimulated with E. coli ssDNA (0 µg/mL, 10 µg/mL, 50 µg/mL, or 100 µg/ml) in the presence or absence of 100 ng/ml LPS for 72 hours. The frequency of proliferating (B220+CFSE-low) B cells was determined by performing flow cytometry analysis. Data, pooled from three independent experiments, are shown as bar graphs (means ±SEM, n = 5). **P<0.01 as compared to LPS or E. coli DNA, and ***P<0.001 as compared to LPS or E. coli DNA. (B) CFSE-labeled naïve B cells were stimulated with E. coli ssDNA (10 µg/mL or 50 µg/ml) in the presence or absence of 100 ng/ml LPS for 72 h. Cells were analyzed by flow cytometry for CD138 surface expression. Representative dot plots of three independent experiments show the percentages of CD138+ plasma cells generated under different culture conditions. (C) Naïve B cells were cultured in media containing E. coli ssDNA (50 µg/ml) with or without LPS (100 ng/ml) for 72 h, and cell culture supernatants were collected for analysis of IL-10 by ELISA. Data, pooled from three independent experiments, are shown as bar graphs (mean ±SEM, n = 4).*P<0.05 as compared to LPS. (TIF) [file pone.0109095.s002.tif]

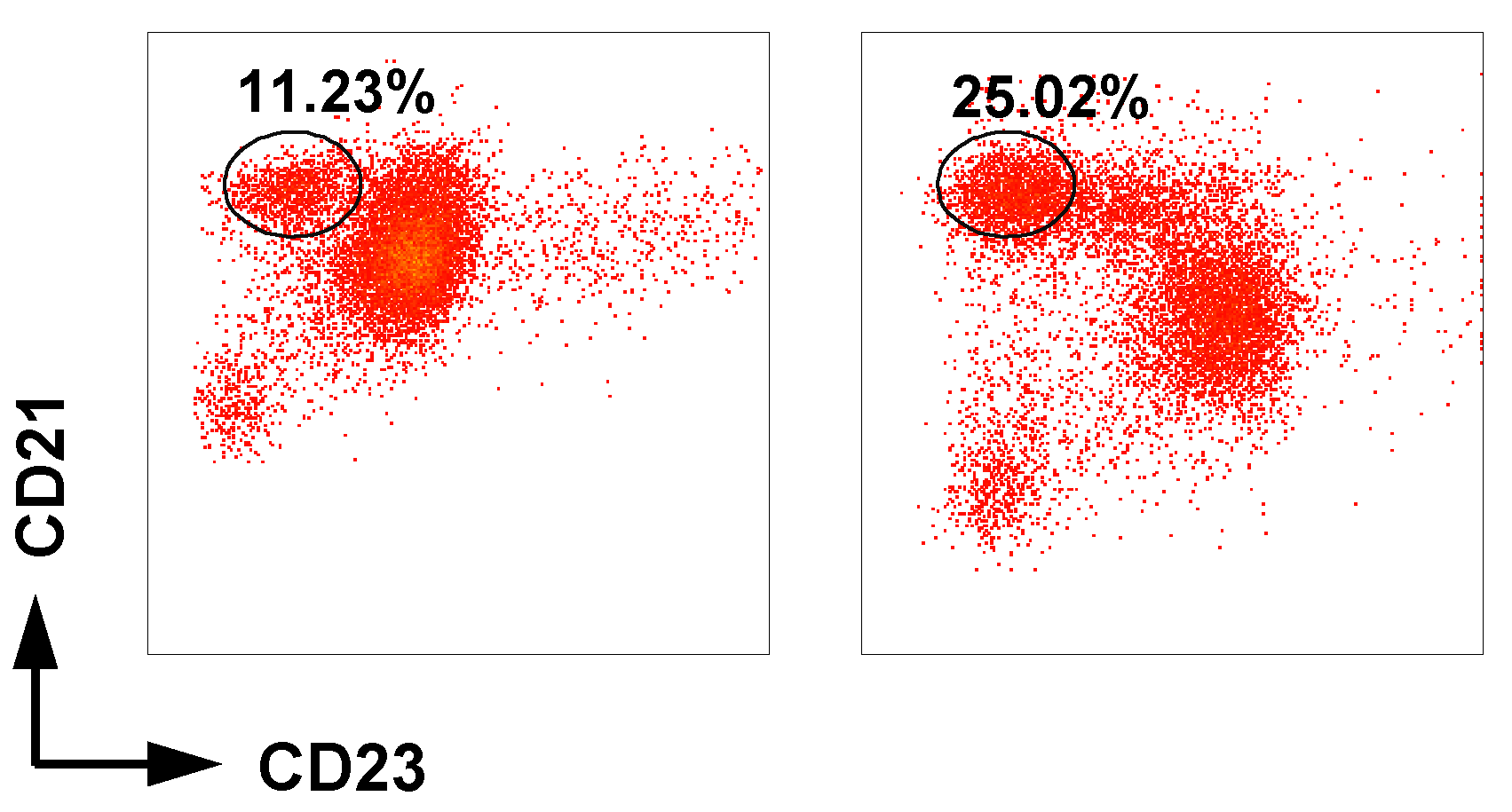

Supplement: Figure S4 — Frequency of MZ B cells in normal and MRL+/+ lupus mice. Splenic B cells were isolated from normal BALB/c and MRL+/+ mice by negative selection using anti-CD43-coated magnetic beads. The frequency of MZ B cells (CD21hi CD23lo) was determined by flow cytometry. (TIF) [file pone.0109095.s004.tif]
